# Anæsthesia by Compression

**Published:** 1859-04

**Authors:** 


*Anæsthesia by Compression.*—According to the *Gazette des Hopitaux*, M. Jaconski has revived the practice of compression as a means of preventing pain. It has been used in the extraction of teeth with entire success. His arrangement resembles that of a double hernial truss, consisting of two pads connected by a steel spring. The spring is passed behind the head, and the pads applied either in the auditory meatus or in front of the ear, and behind the rami of the jaws.

